# Optimization of Chitosan and Cellulose Acetate Phthalate Controlled Delivery of Methylprednisolone for Treatment of Inflammatory Bowel Disease

**DOI:** 10.15171/apb.2017.025

**Published:** 2017-06-30

**Authors:** Swati Jagdale, Apoorva Chandekar

**Affiliations:** Department of Pharmaceutics, MAEER’s Maharashtra Institute of Pharmacy, MIT Campus, S. No. 124, Kothrud, Pune 411038, Savitribai Phule Pune University, India.

**Keywords:** Methylprednisolone, Colon, Targeting, Press coated, Tablet, Chitosan

## Abstract

***Purpose:*** Inflammatory bowel disease (IBD) is a chronic, relapsing and often life-long disorder. The best way to tackle IBD is to develop a site targeted drug delivery. Methylprednisolone is a potent anti-inflammatory steroid. The relative potency of methylprednisolone to hydrocortisone is at least four is to one. The aim of the present research was to develop a colon targeted drug delivery for treatment of IBD.

***Methods:*** Compression coated drug delivery system was designed and optimised. Core tablet contained drug, croscarmellose sodium (CCS-superdisintegrant), avicel (binder) and dicalcium phosphate (diluent). Design of experiment with 3^2^ factorial design was applied for optimization of compression coated delivery. Chitosan and cellulose acetate phthalate were chosen as independent variables. Swelling index, hardness and % drug release were dependant variables.

***Results:*** Core tablet (C5 batch) containing 2.15% CCS showed disintegration in less than 10sec. FTIR, UV and DSC study had shown absence of any significant physical and chemical interaction between drug and polymers. F8 was found to be optimised formulation. F8 contained 35% chitosan and 17.5% cellulose acetate phthalate. It showed drug release of 86.3% ± 6.1%, hardness 6.5 ± 1.5 and lag time 7 hrs. Simulated media drug release was 97.51 ± 8.6% with 7.5 hrs lag time. The results confirmed that the lag time was highly affected by the coating of the polymers as well as the concentration of the superdisintegrant used in core tablet.

***Conclusion:*** In-vitro and in-vivo results confirmed a potential colon targeted drug therapy for treatment of IBD.

## Introduction

Oral drug delivery is always the preferred form of delivery. The main problem with oral drug delivery is first pass metabolism. This becomes a major problem when drug needs to reach to the lower part of the gastrointestinal tract (GIT). To overcome this problem, targeted drug delivery systems are being developed. These formulations are designed to reach to the specific site to deliver drug.^1^ Controlled delivery technology is divided in two subtypes as programmed and triggered release technology. Programmed release depends largely on the build-in program to release the drug rather than depending on interaction with the environment in a certain gastrointestinal segment (site specific target) or with a disease specific target. Examples of release profiles in this category are prolonged or delayed release. Triggered release technologies are the one where release is initiated upon interaction of the delivery system with a site specific target as pH, pressure or microbial flora. Since there is no influence of disease specific factors, this profile is often called site specific release.^2^ In last few years the development of colon targeted drug delivery has received an increasing interest not only for a better treatment of specific local pathologies but also for the systemic therapy of both conventional and labile molecules. Colon targeted drug delivery system can be used to treat a number of diseases like colitis, Crohn’s disease and colorectal cancer.^3^ Especially those drugs that degrade in the hostile environment of the stomach and intestine can be protected from degradation and delivered directly to the colon. A large number of anti-asthmatics are delivered through this route.

Various approaches have been used in colon targeted drug delivery. Few include pH dependent system, time dependent system, pH and time dependent system and microbiologically triggered system. Newly developed techniques include pressure controlled drug delivery system, CODES ^™^and osmotic controlled drug delivery system.^4,5^ Pulsatile drug delivery systems are gaining a lot of interest now days. These systems are designed according to the circadian rhythm of the body. They deliver the drug at the specific time as per the pathophysiological need of the disease, resulting in improved patient compliance and therapeutic efficacy.^6,7^

Literature survey has indicated that work has been carried out to target colon with delivery systems as time delayed capsule for diltiazem hydrochloride,^8^ pulsatile delivery of theophylline,^9^ flurbiprofen^10,11^ and simvastatin,^12^ piroxicam,^13^ compression coating of 5-aminosalicylic acid matrix tablets,^14^ fenoprofen calcium compressed coated tablets,^15^ pH dependent delivery system of lercanidipine hydrochloride^16^ etc.

Methylprednisolone is a potent anti-inflammatory steroid. It has greater anti-inflammatory potency than prednisolone. Even have fewer tendencies than prednisolone to induce sodium and water retention. The relative potency of methylprednisolone to hydrocortisone is at least four is to one. It belongs to BCS class II drug. Absolute bioavailability of methylprednisolone in normal healthy subjects is high i.e 82% to 89% after oral administration. The mean elimination half-life for methylprednisolone is in range of 1.8 to 5.2 hrs. Drug undergo high hepatic metabolism.^17-20^ Literature study showed that dosage forms designed for methylprednisolone include freeze-dried product, immediate release (IR) tablet, immediate release capsule, microparticles in treatment of arthritis and pH sensitive hydrogels.^21,22^ Commercially it is available as immediate release (IR) tablet. IR tablet has drawback of fluctuation to attain peak plasma concentration, variability in gastric emptying time, dose dumping, extensive first pass effect and low bioavailability. For colon diseases development of targeted drug delivery will release the drug at the site. This will minimize the side effects of the conventional dosage form and hence will improve the bioavailability and patient compliance. In the present research work methylprednisolone was chosen as a drug to target colon for treatment of IBD and colitis. The aim of the research was to give a site and time controlled drug delivery to colon. To achieve this aim, a compression coated drug delivery system containing chitosan and cellulose acetate phthalate was designed and optimized.

## Materials and Methods

Methylprednisolone, chitosan, cellulose acetate phthalate (CAP), avicel, croscarmellose sodium (CCS), dicalcium phosphate (DCP), crospovidone, talc and magnesium stearate were purchased from Analab Fine chemicals, Mumbai.

### Characterization of drug

Drug was characterized for melting point (melting point apparatus - LABIN), FTIR spectra (Varian 640 IR) in the wavelength region of 4000-400 cm^-1^ with diffusion reflectance principle and differential scanning calorimeter (DSC-60) at a temperature range of 50-300 °C with rate of 10 °C per minute in nitrogen atmosphere.

Solution of the drug in three different media (0.1N HCl, 7.4 pH phosphate buffer and 6.8 pH phosphate buffer) was scanned by UV-Visible spectrophotometer (UB Varian Carry 100) and λ _max_ was reported. Calibration curve in three media were recorded. The analytical method was validated for accuracy, inter-day and intra-day precision, linearity, limit of detection and limit of quantification. Stability of the drug was checked in all three dissolution media for duration of 24 hrs.

### Drug-polymer interaction study

Drug alone, drug with polymers (chitosan and CAP) were mixed in 1:1 ratio. They were kept in stability chamber (Thermolab Scientific Equipments, 90/90/130 litres) for one month at accelerated stability conditions at 40 ± 2°C / 75 ± 5% RH. At the end of month, samples were analysed for FTIR, DSC and UV spectra.

### Formulation of core and compression/press coated tablet

Core tablet were prepared using crospovidone as superdisintegrant in concentration of 1-5%. But those tablets lacked in hardness and had broken easily. Hence, crospovidone was replaced by croscarmellose sodium (CCS). Dicalcium phosphate (DCP) was used as diluent and avicel as binder. Tablets were compressed on Cadmac, Rimeck Minipress (8 station) machine using 6 mm round concave punch. Formulations were prepared with different concentration of CCS (1.5-8.5%) as shown in Table 1. Batch C5 was found to be most suitable formulation based on disintegration time and hardness.

**Table 1:** Formulation of core tablet

**Ingredients (mg)**	**C1**	**C2**	**C3**	**C4**	**C5**	**C6**
Methylprednisolone	8	8	8	8	8	8
Avicel ph 101	50	58	61	50	51	50
CCS	6	1	1	6	1.5	2
DCP	6	3	-	6	9.5	10

Total weight = 70 mg

Chitosan and CAP were chosen as polymer for outer coating of core tablet. Talc and magnesium stearate were added along with polymers as lubricant. DCP was dded as a diluent in the outer coat. At initial stage individual polymers were tried in different concentration. Individual polymer failed to maintain its integrity till it reaches to third media. Therefore for further study combination of polymer were tried. Chitosan was used in concentration range of 50-10 % and CAP in 10-50 %. To manufacture compression coated tablet direct compression method was used. 50% of coating mixture was first placed in the die cavity. Core tablet was then placed above this mixture. Then remaining quantity of coating mixture was added in die cavity to cover the core tablet. It was compressed on Cadmac, Rimeck Minipress compression machine (9 mm flat and plain punch) to obtain final weight of the tablet 200 mg with hardness 4-7 kg/cm^2^.^23^ Preliminary trials had shown that 40% chitosan and 20% CAP crossed first two media and enter intact in third media in the dissolution study.

### Design of experiment (DOE)

3^2^ factorial design was used as a tool to optimize the formula for coating polymer. In this design 3 levels and 2 factors were used. The factors were concentration of chitosan and concentration of CAP. The coded levels were +1, 0 and -1.^24,25^ Factorial batch formulations were prepared as shown in Table 2. Manufacturing method is same as discussed above in compression/press coated tablet.^24,25^

**Table 2:** Formulation of compression coated tablet

**Batch code**	**Chitosan (mg)**	**CAP (mg)**	**Talc (mg)**	**Mg. Stearate ( mg)**	**Core Tablet (mg)**	**DCP (mg)**
F1	80 (+1)	40 (+1)	1	1	70	8
F2	80 (+1)	35 (0)	1	1	70	13
F3	80 (+1)	30 (-1)	1	1	70	18
F4	75 (0)	40 (+1)	1	1	70	13
F5	75 (0)	35 (0)	1	1	70	18
F6	75 (0)	30 (-1)	1	1	70	23
F7	70 (-1)	40 (+1)	1	1	70	18
F8	70 (-1)	35 (0)	1	1	70	23
F9	70 (-1)	30 (-1)	1	1	70	28

Total weight = 200 mg; CAP: cellulose acetate phthalate; DCP: Dicalcium phosphate; Mg Stearate: Magnesium Stearate

### Evaluation of core tablet: 

Core tablet was evaluated for thickness (Vernier calliper), hardness (Monsanto hardness tester), friability (Roche friabilator -Veego), uniformity of content (UV- Visible spectrophotometer), weight variation (digital electronic balance) and disintegration time (digital tablet disintegration apparatus -Veego) at 37 ± 2°C in 900 ml phosphate buffer pH 6.8.

*In- vitro* dissolution study was carried out in 900 ml pH 6.8 phosphate buffer (PBS) at 37 ± 0.5°C in USP dissolution apparatus type II (Electro Lab, Model TDT-08L). Rotation speed was maintained at 50 rpm. Samples from this solution were withdrawn at time interval and analysed at λ max.

### Compression /Press coated tablet

Evaluation of powder flow properties was carried out by measuring angle of repose (fixed funnel method), bulk density and tapped density (digital bulk density apparatus - Bio Techno Lab, Make Lab HOSP).Compressibility index and Hausner’s ratio was calculated.

Evaluation of coated tablet for tablet thickness, friability, drug content, weight variation and hardness were carried out in similar manner as mention in evaluation of the core tablet.

To determine swelling index for tablet, it was immersed in a flask of USP dissolution test apparatus containing 250 ml of medium at 37°C (for first 2 hrs in pH 1.2 HCl, next 3 hrs in pH 7.4 PBS and lastly in pH 6.8 PBS for 3 hrs). At specific time interval, the swollen tablets were withdrawn from the medium and weighed. Light blotting with a filter paper was used for removal of excess surface water. Study was performed in three replicates. The swelling behavior of the coated tablet was calculated using Eq.1

SI= [W_t_ -W_0_] / W_0_ ×100 Equation 1

Where, SI is swelling index of the tablet, W_t_ is the weight of tablet after appropriate time interval t in respective media and W_0_ is the initial weight of dry tablet.

For* in- vitro* dissolution study the tablets were placed in USP dissolution apparatus type II. To simulate the pH changes along the GI tract, three dissolution media were used. Recent studies with sensitive and reliable equipment contradict the traditional view and provide evidence of a decrease in pH at the gastrointestinal region between the ileum and the colon. Apparently the colon has a lower pH value (6.5) than that of the small intestine (7.0–7.8).^9^ Radio telemetry showed highest pH (7.5 ± 0.5) in the terminal ileum. On entry into the colon, the pH drops to 6.4 ± 0.6. The pH in the mid colon is 6.6 ± 0.8 and in the left colon 7.0 ± 0.7.There is fall in pH on entry into the colon due to the presence of short chain fatty acids arising from bacterial fermentation of polysaccharides.^4^Considering these reported references in present study first media used was pH 1.2 HCl for 2 hrs (average gastric emptying time is about 2 hrs). Second media was pH 7.4 phosphate buffer solution for next 3 hrs (average small intestinal transit time is about 3 hrs). Lastly the study was carried out in third media i.e. pH 6.8 phosphate buffer solution (colon) until the tablet burst.^4,8,9,16^ Samples were collected till 8 hrs at specific time interval and analysed by UV spectrophotometer. Percent drug release was calculated.

For dissolution study in simulated media F7 and F8 batches chosen. Simulated media were based on sequential pH change method as gastric fluid pH 5, intestinal fluid pH 5.8 and colonic fluid pH 7. The composition of the media was prepared and used in present study was as reported by Marques et al.^26^

Stability study was carried for the optimized batch F8 at long term (30°C ± 2°C/65% RH ± 5% RH) and accelerated conditions (40±2°C/75±5% RH) over a period of three months according to ICH guidelines in stability chamber (Thermo Lab Scientific Equipments). At the end of three months and in between time intervals for long term stability study, tablets were withdrawn from the stability chamber and examined for physical evaluation and drug content.

### Statistical analysis of data

*In-vitro* release kinetics was carried by PCP DISSO.v2.08

Similarity factor was calculated using BIT software. Similarity study was carried out in between marketed tablet of methylprednisolone (Medrol 8 mg, Pfizer Italia) and optimized formulation F8.

Design expert software version 9.0 was used to obtain ANOVA analysis and response surface graphs for factorial design.

### In-vivo X-ray imaging placebo study

*In-vivo* X-ray imaging placebo study was performed on three healthy human volunteers using X-ray generating unit. Optimized batch F8 was evaluated. Methylprednisolone was replaced with radio opaque agent, barium sulphate. The core tablet contained barium sulphate (8 mg), CCS (1.5 mg), Avicel (51 mg) and DCP (9.5 mg). For coated tablet chitosan (70 mg), CAP (35 mg), DCP (23 mg), talc (1 mg) and magnesium stearate (1 mg) were used. The study was carried out as per World Medical Association guidelines and Helsinki ethical principle. The aim was only to check the position of optimized formulation in body. The study does not involve any blood or any other kind of sampling. Informed consent of human volunteer was taken before participation. The study was carried out under supervision of expert radiologist and physician. For placebo study volunteers aged between 24-26 years and weighing 55-60 Kg were selected. Each volunteer after an overnight fasting was ingested with tablet orally with 200 ml water. The volunteers were served with breakfast at 2 hrs and lunch at 4 hrs after the administration of the tablet. The tablets were visualized using X-ray technique. Abdominal radiographs were taken after 1, 2 and 5 hrs in all volunteers.^27,28^

## Results and Discussion

### Characterization of drug

**Figure 1:**
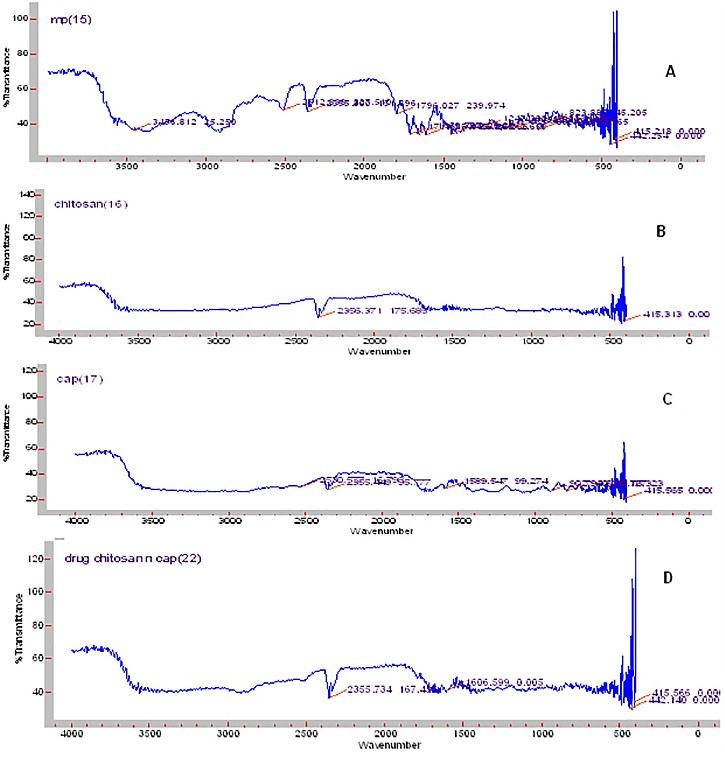
FTIR spectra for (A) Methylprednisolone, (B) chitosan, (C) CAP and (D) Formulation

Melting point of drug was obtained at 231° C which was matching with the reported literature data [18].This proved purity of drug. FTIR spectra (Figure 1A) showed sharp peaks at 1450 cm^-1^ and 1375 cm^-1^ indicating CH_3_ (bend), peak at 1680-1600 cm^-1^ indicated C=C (alkenes), peak at 1300-1000 cm^-1^ indicated C-O (alcohol), at 1725-1705 cm^-1^ indicated C=O (ketone), broad absorption near 3400-3300 cm^-1^ indicated O-H (alcohol). All these peaks indicated presence of drug in pure form matching with standard values reported in the literature.^29^

**Figure 2:**
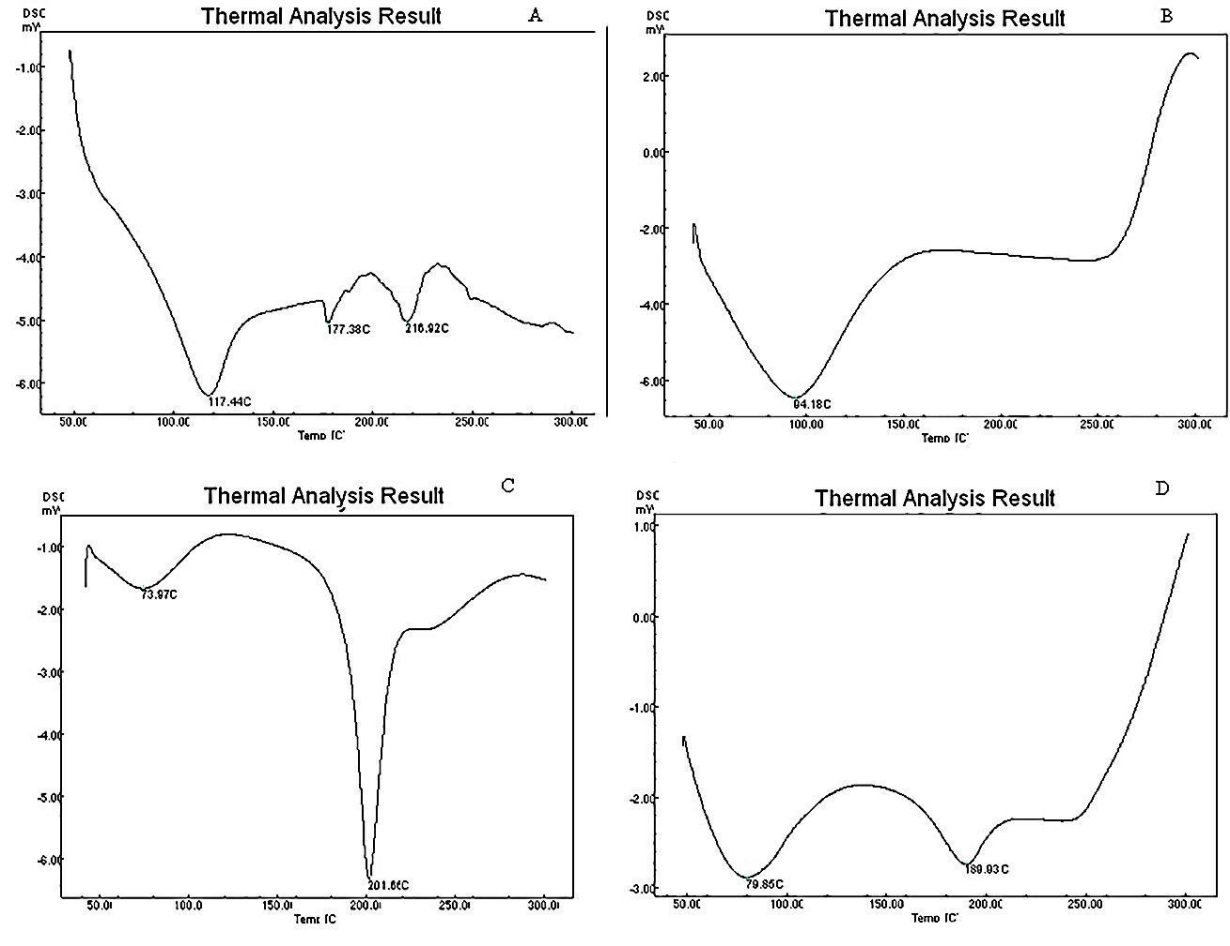
DSC graph of (A) Methylprednisolone, (B) chitosan, (C) CAP and (D) Formulation.

The DSC graph for methylprednisolone (Figure 2A) showed sharp peaks at 117.4°C, 177.38° C and 216.9°C.

UV spectroscopic study showed λ max at 246.13 nm. It was found that the drug follows Beer-Lambert’s law. For accuracy study, percent recovery for 80% was 98.92%, for 100% it was 99.36% and for 120% it was 99.19%. From the data obtained from interday as well as intraday study, the method was found to be precise. The drug solution was found to be linear in the range on 5-25µg/mL. LOD and LOQ were found to be 0.157µg/mL and 0.549µg/mL respectively.

### Drug- polymer interaction study

As shown in Figure 1A, FTIR spectra of drug showed peaks at 1450 cm^-1^and 1375 cm^-1^, 1680-1600 cm^-1^, 1300-1000 cm^-1^, 1725-1705 cm^-1^and broad absorption near 3400-3300 cm^-1^. Chitosan (Figure 1B) showed peak at 2356 cm^-1^ and 415 cm^-1^. CAP (Figure 1C) showed peak at 415 cm^-1^,2350 cm^-1^, 2355 cm^-1^, 1589 cm^-1^,902 cm^-1^ and 796 cm^-1^. Formulation (Figure 1D) showed peaks at 415 cm^-1^,1600 cm^-1^ and 2355 cm^-1^. Few peaks of drug as well as polymer may have merge due to polymer interlinking and engulfing of drug in matrix. As the major peak for drug and polymer remains unchanged it can be said that there was no significant physical and chemical interaction between drug and polymer.

DSC graph of pure methylprednisolone (Figure 2A) showed peaks at 117°, 177° and 216°C. Chitosan (Figure 2B) showed peak at 94° C while CAP (Figure 2C) showed peaks at 73° and 201°C . Formulation F8 (Figure 2D) on the other hand showed peaks at 79°and 189° C. Some peaks of CAP and methylprednisolone did not shift significantly while the remaining peak of CAP, methylprednisolone and chitosan were not present. The shift in the peak can be explained mainly due to polymer interlinking and engulfing of the drug in the polymer matrix.

UV spectra had shown that λ _max_ for the drug remained same with polymer as well as in the formulation. As there was no shift or change in λ _max,_ it indicated absence of any kind of physical and chemical interaction between drug with polymer and excipients.

### Formulation of core and coated tablet

In manufacturing for core tablet crospovidone was used as superdisintegrant at initial stage but the batches failed to meet disintegration criteria. Hence croscarmellose sodium was used. Also, lactose was used as diluent in earlier trial but the tablets did not have enough hardness, Hence, it was replaced by DCP. C5 was found to be most suitable batch with croscarmellose sodium as superdisintegrant which was used further for compression coating.

Preliminary trial batches for coated tablet had shown hardness in the range of 4.5-7 kg/cm^2^. Individual polymers were tried but those tablets broke even before reaching the second media. The concentration of the polymers were varied in 50 -10% of chitosan and 50 -10% of CAP. It was observed that tablet containing higher concentration of chitosan did not disintegrate after 24 hrs. Whereas tablet containing higher concentration of CAP did not had enough hardness to hold the tablet till it reaches to third media. The tablet containing 40% chitosan and 20% CAP was found to be suitable as it reached to third media.

Core tablet C5 showed thickness in the range of 2 ± 0.01mm, hardness 1.5 ± 0.02kg/cm^2^, diameter 6 ± 0.1mm, friability 0.47 ± 0.1%, uniformity of content 99.26 ± 4.3%, uniformity of weight 70 ± 0.2 mg, disintegration time 5 ± 3 sec and percent drug release 99.62 ± 5.6%.

### Design of experiment

The powder blend for all batches had shown angle of repose in range of 25-30°, bulk and tap density 0.48-0.69, compressibility index 11-20% and Hausner’s ratio 1.13-1.20. All these results showed good flow properties for the powder blend as reported in literature.^30^

For F1 to F9 batches, hardness was found in the range of 5- 6.5 kg/cm^2^, weight variation 199-202 mg, friability 0.37 - 0.52%, drug content 97- 99%, thickness was around 0.2 mm and diameter was around 9 mm. Summary for all evaluation parameters is as shown in Table 3.

**Table 3:** Post-compression parameters evaluation for coated tablet

**Batch code**	**Hardness (kg/ cm** ^ 2 ^ **)**	**Weight variation**	**Friability (%)**	**Drug Content (%)**	**Thickness (mm)**	**Diameter (mm)**
F1	5.5 ± 1.4	200.1 ± 1.2	0.52 ± 0.01	98.57 ± 1.2	0.2 ± 1.6	9 ± 1.3
F2	5 ± 1.3	200.5 ± 1.6	0.49 ± 1.1	97.06 ± 1.6	0.2 ± 1.1	9 ± 1.5
F3	5 ± 1.2	201.4 ± 1.5	0.46 ± 1.2	98.97 ± 1.9	0.2 ± 1.2	9 ± 1.6
F4	5.5 ± 1.4	199.3 ± 1.6	0.42 ± 1.3	98.50 ± 1.6	0.2 ± 1.6	9 ± 1.5
F5	6 ± 1.5	199.7 ± 1.2	0.37 ± 1.5	98.43 ± 1.7	0.2 ± 1.7	9 ± 1.9
F6	5.5 ± 1.0	199.8 ± 1.8	0.47 ± 1.4	97.93 ± 1.3	0.2 ± 1.4	9 ± 1.8
F7	6 ± 1.3	200.2 ± 1.6	0.41 ± 1.8	99.4 ± 1.4	0.2 ± 1.8	9 ± 1.3
F8	6.5 ± 1.5	200.2 ± 1.7	0.38 ± 1.6	99.25 ± 1.6	0.2 ± 1.6	9 ± 1.2
F9	5.5 ± 1.4	202.3 ± 1	0.39 ± 1.2	98.06 ± 1.2	0.2 ± 1.2	9 ± 1.1

**Figure 3:**
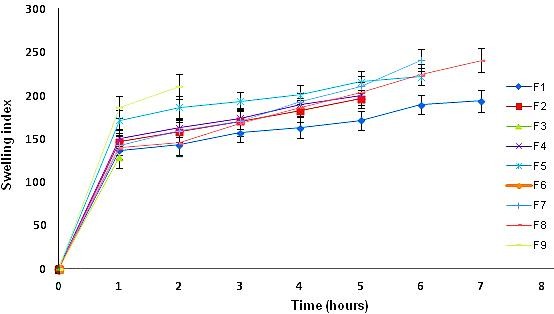
Swelling indices for F1-F9 batches

Polymers played a major role in the swelling property of the tablet. Chitosan and CAP are both hygroscopic polymers. On entering the media, both polymers had shown swelling property. Direct relation was found between swelling index and lag time. The swelling pattern for batches can be seen in Figure 3. The tablet having maximum swelling index had shown highest lag time. The swelling capacity of CAP played a major role in swelling index of tablet. The tablet containing higher concentrations of CAP had shown maximum swelling as compare to low concentration. Those tablets with low concentration of CAP had broken at faster rate. F1 and F8 batches showed maximum swelling whereas F6 had broken in half an hour only. Swelling capacity of polymer HPMC had shown similar role for reported work of diltiazem, lercanidipine and flurbiprofen.^8,11,16,25^

**Figure 4:**
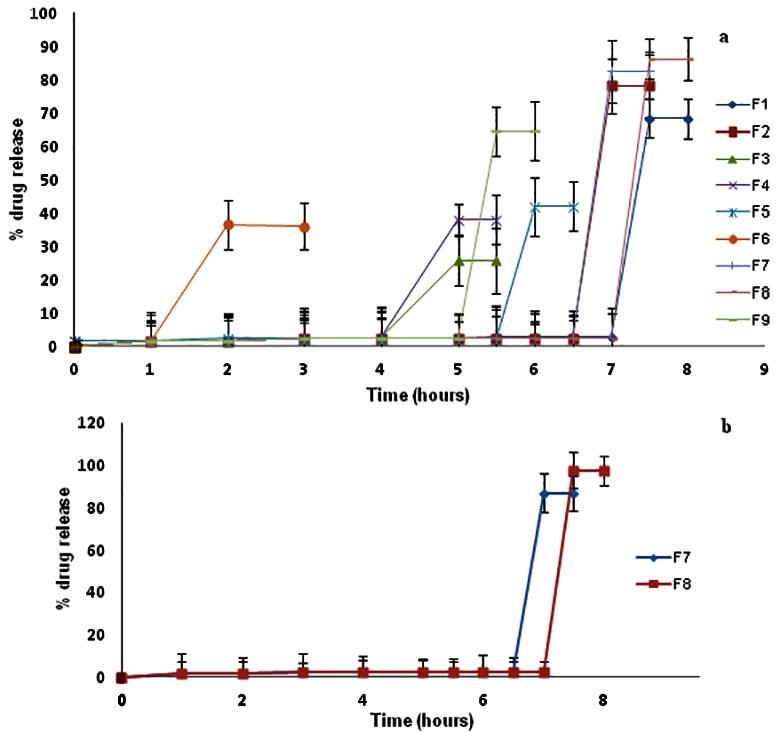
Dissolution profile **a:** F1-F9 batches; **b:** F7 and F8 in simulated media

From* in-vitro* dissolution study, it was observed that both the polymer starts swelling after coming in contact with the media. After reaching their maximum swelling capacity, the outer layer of the tablet contacting polymers get rupture which allow core tablet to come outside .Since the core tablet contains superdisintegrant, it immediately broke down and released the drug in outer media. It can be seen (Figure 4a) that F6 and F9 disintegrated before the time it can reach to third media that is colon (before 5 hrs). F6 and F9 contain low concentration of CAP. This indicated that the concentration of CAP played an important role in bypassing first two media. *In-vitro* dissolution study proved that minimum 17.5% CAP was required in the tablet so as to reach to the colon. Batches F7 and F8 showed the maximum drug release (≥ 90%) in 7-8 hrs.^25,31^

Batches F7 and F8 were studied in simulated media. Batch F8 (35% chitosan and 17.5% CAP) showed maximum drug release of around 97.51 ± 5.6% (Figure 4b).

After 3 months for batch F8, evaluation parameters were as thickness (0.2 ± 0.04 mm), diameter (9 ± 0.5 mm), hardness (6 ± 1.4 kg/cm^2^), weight variation (199.54 ± 1.6 mg), friability (0.34 ± 1.1%), percent drug release (85.43 ± 5.9%) and percent drug content (98.16 ± 1.9%). There was no significant change in the values before and after indicating the stability of the formulation.

Batch F8 showed zero order release. It indicated that the dosage form releases same amount of drug in a given unit of time.^32^

### Response surface plots

Response surface plots were used to determine the effect of independent variables on the dependent variables. Independent variables were concentrations of both the polymers whereas dependent variables were swelling index, hardness and percent drug release. Response surface plots were derived from design expert software version 9.0.3.1. It was seen that swelling index was most significant between 3-5 hrs whereas percent drug release and hardness were not found to be that significant factors.

Equation for swelling index at 3 hrs:

Swelling index = +184.89 – 2.17A + 83.33B – 3.25AB Equation 2

Equation for swelling index at 5 hrs:

Swelling index = +210.67- 7.83A + 97.00B – 10.00AB Equation 3

Where, A = chitosan and B = CAP

ANOVA results indicated that swelling index was the only significant factor. This indicated that change of media played an important role for swelling of the tablet. The media was changed first after 2 hrs and then after 5 hrs. Thus, when the tablet was in the second media the swelling was most dominant. When the tablet reached to the third media the swelling got continued but it did not have that much significant effect as compared with first two media.

**Figure 5:**
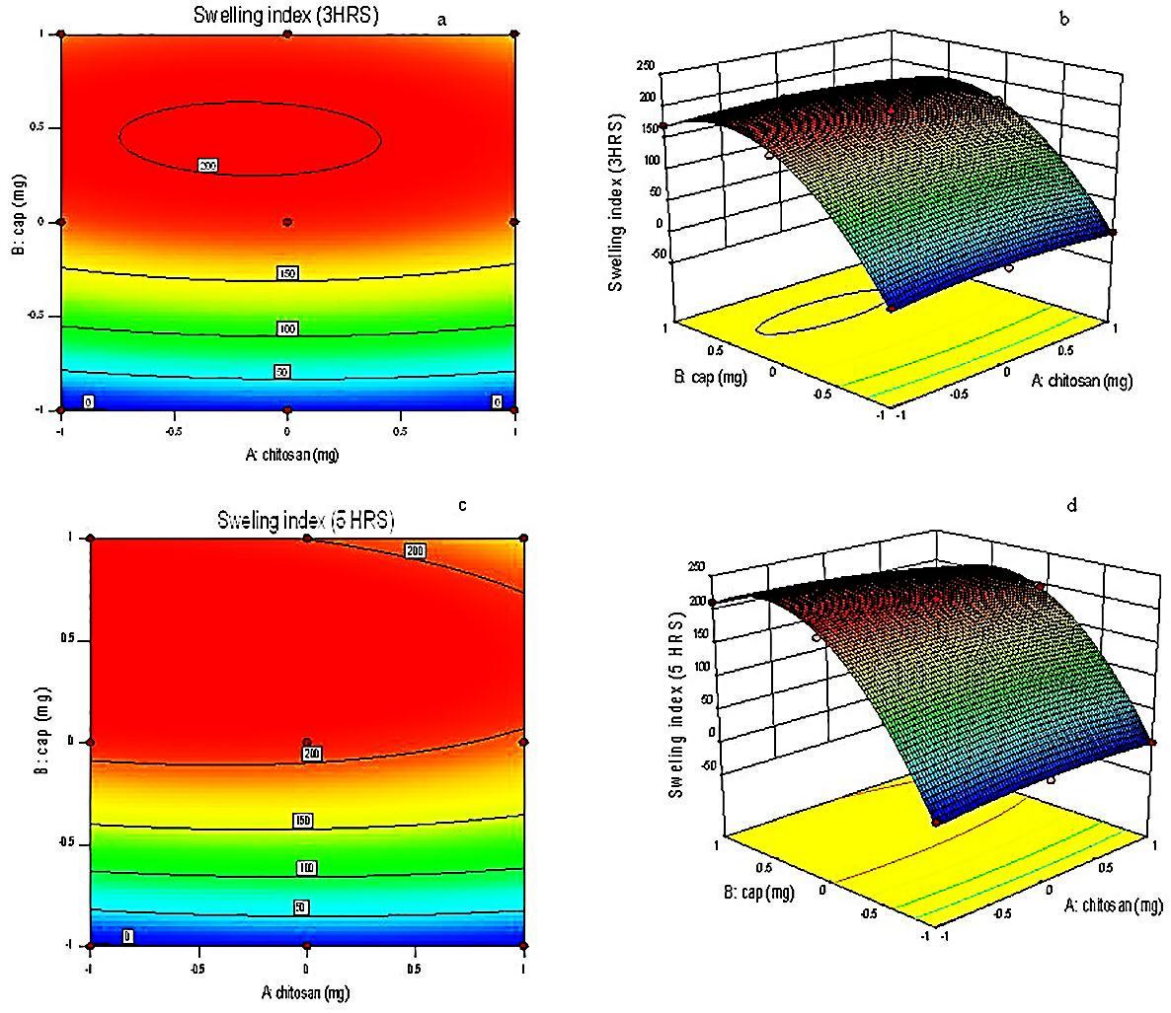
**a, c**: Contour plot showing the influence of chitosan and CAP on swelling index at 3 hrs and at 5 hrs **b, d**: Response surface plot showing the relationship between various levels of chitosan and CAP at 3 hrs and at 5 hrs

Response surface and contour plots of the respective responses were generated as shown in Figure 5. Table 4a indicates the ANOVA data.^33^

**Table 4a:** ANOVA results

**Response model**	**Sum of squares**	**df**	**Means square**	**F value**	**P value**	**R** ^2^	**Adequate precision**
Swelling index at 3 hrs	59769.36	5	11953.87	186.59	0.0006	0.9968	29.009
Swelling index at 5 hrs	80694.67	5	16138.93	328.62	0.0003	0.9982	37.691

From Eq. 2; for swelling index at 3 hrs, it was found that CAP showed positive effect on swelling index. Chitosan showed significantly less effect on swelling index as compared to CAP. The p-value for swelling index at 3 hrs was found to be 0.0006 which was less than 0.0500.This indicated that the model was highly significant.

From Eq. 3; for swelling index at 5 hrs, it was found that again CAP showed positive effect while chitosan showed negative effect. The difference in the values between 3 and 5 hrs was not much large.

Predicted optimized batch by design expert was F8 batch. Predicted results as shown in Table 4b indicated validation of design expert software.

**Table 4b:** Validation Data

**Composition**			**Predicted value**		**Observed value**		**Standard deviation**		**SEM (Std. error of mean)**	
Polymer	Coded value	Actual value (mg)	SI at 3 hrs (%)	SI at 5 hrs (%)	SI at 3 hrs (%)	SI at 5 hrs (%)	SI at 3 hrs	SI at 5 hrs	SI at 3hrs (%)	SI at 5hrs (%)
Chitosan	-1	70	176.22	210	167.9	203.4	8.00405	7.00793	5.96587	5.2234
CAP	0	35								

The similarity factor (f_2_) obtained was 14. The value indicated that marketed formulation (immediate release) and the designed formulation were not similar in their release pattern. As the design dosage form was to be targeted to colon, this gave indication that the designed can reach to the colon.^34^

### In-vivo X-ray imaging (placebo) study

**Figure 6:**
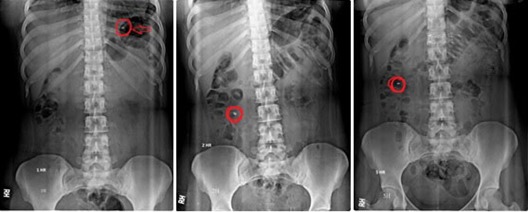
X-ray images for placebo batch F 8 at 1hr, 2hrs and 5 hrs

Radiographs of human volunteers were taken after a specified time interval. *In - vivo* X-ray imaging placebo study for colon targeted delivery of methylprednisolone in human volunteers had shown that the tablet did not float in stomach. The tablet even did not break down in stomach and small intestine. Tablet remains intact. Tablet also did not adhere to any part of the gastrointestinal tract i.e. stomach and small intestine. From Figure 6 it can be seen that the tablet can be located in the ascending part of colon after 5hrs of ingestion. This indicated that the optimized formulation in body will have the capacity to pass stomach and small intestine and enter in third media which is colon. This gave a positive assurance that the optimized delivery will give targeted drug delivery to colon.^35^

## Conclusion

An attempt had been made to optimize a colon targeted drug delivery containing polymer chitosan and cellulose acetate phthalate. The main objective was to avoid first pass metabolism of the drug and to give a therapy for colon diseases. Direct compression technique was used for compression coated delivery. Design of experiment was applied for optimization of coating layer. F8 was the optimized batch. ANOVA results indicated that swelling index was the most significant factor in controlling the lag time and hence release of drug. CAP played most important role in swelling.* In-vitro* as well as *in –vivo* study indicated presence of delivery in third media indicating protection of the drug from being released in the upper region of GI system, i.e. stomach and small intestine. Thus F8 formulation can act as potential for treatment of colon diseases that can achieve maximum therapeutic efficacy.

## Acknowledgments

The authors are thankful to Savitribai Phule Pune University, Maharashtra, India for providing financial assistance under Board of College and University Development *(BCUD)* Project Grant for the present research work. The authors are thankful to SKN General Hospital and Medical College, Narhe, Pune, for providing facility to carry *In-vivo* x-ray imaging (placebo) study. Authors are also thankful to Dr. B. S. Kuchekar, Principal and management of MAEER’s Maharashtra Institute of Pharmacy, Pune for providing all the facilities to carry out the research work.

## Ethical Issues

The study was carried out as per World Medical Association guidelines and Helsinki ethical principle.

## Conflict of Interest

The authors declare no conflict of interests.
